# Case of Strangulated Congenital Hernia; Occurring in a Child, Six Weeks Old, and Requiring Operation

**Published:** 1845-08

**Authors:** James Long

**Affiliations:** Surgeon to the South Dispensary, and Lecturer on Anatomy, Liverpool


*Case of Strangulated Congenital Hernia; occurring in a Child, six weeks old, and requiring Operation.* By JAMES LONG, Esq., Surgeon to the South Dispensary, and Lecturer on Anatomy, Liverpool.—On the 10th of May I was requested to see a male child, born on the 29th of March, and consequently six weeks old; he had cried all night, had passed neither motion nor flatus, strained frequently, and had vomited a yellowish looking matter. I found a scrotal hernia on the right side, the size of a hen's egg; the bulk of this, after a little trouble, was reduced, but a portion, the thickness of the little finger, occupying the inguinal canal, and having the testicle at the bottom, remained; this was exceedingly tender to the touch, and could not, by any justifiable effort I could use, be returned. The warm bath, cold applications, enemata, and the forced injection of warm water, through a long tube, failed to procure either a motion or the evacuation of flatus, or to enable me to return the intestine. These means had been employed during that day, and the greater part of the following, and tympanites and tenderness of the abdomen commencing, I proposed the operation, and requested the presence of Mr. Halton. He fully concurred in the propriety of resorting to it.

It was performed in the usual way, on the 11th. It was my intention, after dividing the stricture, to return the intestine without opening the sac, but finding this impossible, I opened the sac which contained a knuckle of intestine of a light port-wine colour, in contact with and adherent to the testicle, by a band of coagulable lymph, which was easily torn through. The stricture being divided, the crying of the child forced down several folds of intestine, the colour of which strongly contrasted with the strangulated portion, and the indented line of demarcation caused by the stricture formed a distinct boundary between the two. The most difficult part of the operation was to reduce the intestine, the forcing efforts and crying of the child being insurmountable until the difficulty was overcome by the occurrence of fainting. Three sutures, compress and bandage, were applied.

The mother, who had been greatly distressed, was directed not to give the breast until the following morning, and in the meantime to have them drawn; the child was allowed nothing but barley water. During the night two motions passed, the first exceedingly fœtid; in the course of the day two more motions. On the evening of the 13th, the sutures were removed; on the 14th, a teaspoonful of castor oil was exhibited; on the 17th the child was quite well, the wound healed, &c., and I discontinued my visits. There is no appearance of the return of the hernia.—*Lond. Prov. Med. and Surg. Journ.*

